# Persistent disparities despite declining carbon monoxide mortality in the United States: a nationwide analysis, 1999–2023

**DOI:** 10.3389/fpubh.2026.1877803

**Published:** 2026-09-22

**Authors:** Wanling Xu, Zheng Li, Yuhang Mu, Xiadong Du, Li Pang

**Affiliations:** 1 Department of Emergency Medicine, The First Hospital of Jilin University, Changchun, Jilin, China; 2 Department of Abdominal Ultrasound, The First Hospital of Jilin University, Changchun, Jilin, China

**Keywords:** carbon monoxide poisoning, health disparities, injury epidemiology, mortality trends, racial and ethnic disparities, urban–rural disparities

## Abstract

**Background:**

Carbon monoxide (CO) poisoning remains a preventable cause of mortality. However, emerging evidence suggests that recent progress in prevention may not be equitably experienced across the population groups in the United States. Long-term national assessments of disparities in CO mortality are inadequate.

**Objectives:**

This study sought to identify trends in CO poisoning mortality, assess disparities, and inform future studies.

**Methods:**

We conducted a descriptive epidemiological study, using national mortality data from the Centers for Disease Control and Prevention Wide-ranging Online Data for Epidemiologic Research (CDC WONDER) for 1999–2023. The study population included adults aged 25 years and older, with 47,160 deaths identified. Age-adjusted mortality rates were calculated, and trends were assessed using joinpoint regression to estimate annual and average annual percent change (APC and AAPC), with 95% confidence intervals (CIs). Analyses were stratified by sex, race and ethnicity, age group, geographic region and urban–rural status. All analyses represent primary use of these data.

**Results:**

National age-standardized mortality declined over the study period (AAPC −3.02%; 95% CI − 4.58 to −1.43), but the decline slowed after 2010. Mortality among Hispanic adults increased substantially after 2016, with signs of trend reversal among non-Hispanic Black adults. Mortality in non-metropolitan areas remained approximately 1.8 times higher than in metropolitan areas, with evidence suggesting that prior declines may have stalled.

**Conclusion:**

Declines in CO poisoning mortality have slowed in the United States, with some evidence of recurrent annual fluctuations, accompanied by widening racial, ethnic, and geographic differences.

## Background

Carbon monoxide (CO) poisoning, an entirely preventable cause of accidental injury, has been a focus of public health intervention in the United States for a long time. Through comprehensive measures such as legislation mandating CO alarms, public safety education and improved safety standards for gas appliances, progress has been made toward prevention. Between 1999 and 2004, the age-adjusted mortality rate for unintentional, non–fire-related CO poisoning declined to 1.5 per million population (1), a milestone often cited as a public health success in preventing injury. More recent estimates indicate that CO poisoning remains a significant cause of preventable mortality in the United States (2).

However, emerging evidence indicates that the benefits of these achievements may not be equitably experienced across population groups. Beneath the overall decline in mortality lies substantial and possibly widening health disparities (3). Macro-social factors—including increased generator use during power outages, linked to extreme weather events (4), energy shortages and deteriorating housing conditions in certain communities (5, 6) have introduced new and unevenly distributed risks. Existing studies are mostly restricted to specific regions and are done over a short period of time, and few have examined national long-term trends with a special focus on equity. To address this gap, we used nationwide mortality data from CDC WONDER for 1999–2023 to examine trends in CO poisoning mortality among adults in the United States. The study aimed to assess disparities across demographic and geographic subgroups and to provide descriptive evidence that could inform future research and prevention planning.

## Methods

### Study context and population

This descriptive study used mortality certificate data from the CDC WONDER database for 1999–2023 to examine CO poisoning–related mortality among adults aged ≥25 years. CO poisoning deaths were defined as those with International Classification of Diseases, Tenth Revision (ICD-10) code T58 (toxic effect of CO) listed as an underlying or contributing cause of death. We used the Multiple Cause of Death data files from CDC WONDER, which capture all causes listed on the death certificate—including both underlying and contributing causes of death. This study was approved by the Medical Ethics Committee of the First Hospital of Jilin University (approval No. 2026-MS-022). The CDC WONDER data used in this study are publicly available and de-identified, and the committee granted a waiver of informed consent. As our analysis was restricted to a single ICD-10 code (T58), each death record was counted only once (Supplementary Table 1). We did not exclude deaths based on intent (e.g., suicide, homicide, undetermined intent) or fire-related status, as our objective was to characterize the overall burden of CO poisoning mortality across all intents and circumstances.

### Data extraction

Data were extracted from annual population counts and included demographic characteristics and geographic region. Demographic variables included sex; age group (25–34, 35–44, 45–54, 55–64, 65–74, 75–84, and ≥85 years); and race and ethnicity, categorized as non-Hispanic White, non-Hispanic Black, Hispanic or Latino and non-Hispanic Other (including American Indian or Alaska Native and Asian or Pacific Islander). Geographic regions followed US Census Bureau classifications (Northeast, Midwest, South, and West). Urban–rural status was defined as metropolitan or non-metropolitan according to the 2013 NCHS Urban–Rural Classification Scheme. Analyses stratified by urban–rural status were restricted to 1999–2020, as this is the most recent period for which the CDC WONDER Multiple Cause of Death database supports urbanization-specific age-adjusted rate calculations.

### Statistical analyses

We calculated crude and age-adjusted mortality rates per 100,000 population with corresponding 95% confidence intervals (CIs). Age-specific mortality rates for each age group were calculated as the number of deaths in that age group divided by the population in that age group, expressed per 100,000 population. These are distinct from age-adjusted rates, which were calculated using the direct method with the 2000 US standard population, consistent with CDC reporting guidelines. Temporal trends were assessed using the Joinpoint Regression Program (version 4.9.0.0; National Cancer Institute) to estimate annual percent change (APC) and average annual percent change (AAPC) in age-adjusted mortality. Joinpoint regression fits a series of connected linear segments on a logarithmic scale and identifies statistically significant changes in slope. We specified a maximum of 5 joinpoints and required a minimum of 2 observations between joinpoints. Model selection was performed using the weighted Bayesian Information Criterion (BIC) with permutation tests (4,500 permutations; two-sided *α* = 0.05) to identify statistically significant changes in trend. Weighted least squares regression was used to account for the variability of population denominators; autocorrelation was not modeled, consistent with standard Joinpoint practice. Trends were considered statistically significant if the permutation test *p*-value was <0.05 (two-sided).

## Results

### Annual trends in age-adjusted mortality from carbon monoxide poisoning

From 1999 to 2023, a total of 47,160 deaths related to CO poisoning occurred among adults aged ≥25 years in the United States (Supplementary Table 2 and Figure 1). Over the study period, the national age-adjusted mortality rate declined significantly (AAPC: −3.02%; 95% CI: −4.58 to −1.43) (Table 1). Joinpoint regression identified distinct temporal phases: a steady decline from 1999 to 2007 (APC: −2.47%; 95% CI: −3.74 to −1.18), followed by a sharp but non-significant decline from 2007 to 2010 (APC: −9.70%; 95% CI: −20.83 to 2.99). From 2010 to 2023, mortality continued to decline, but at a slower rate (APC: −1.75%; 95% CI: −2.50 to −1.00) (Table 2).

**Figure 1:**
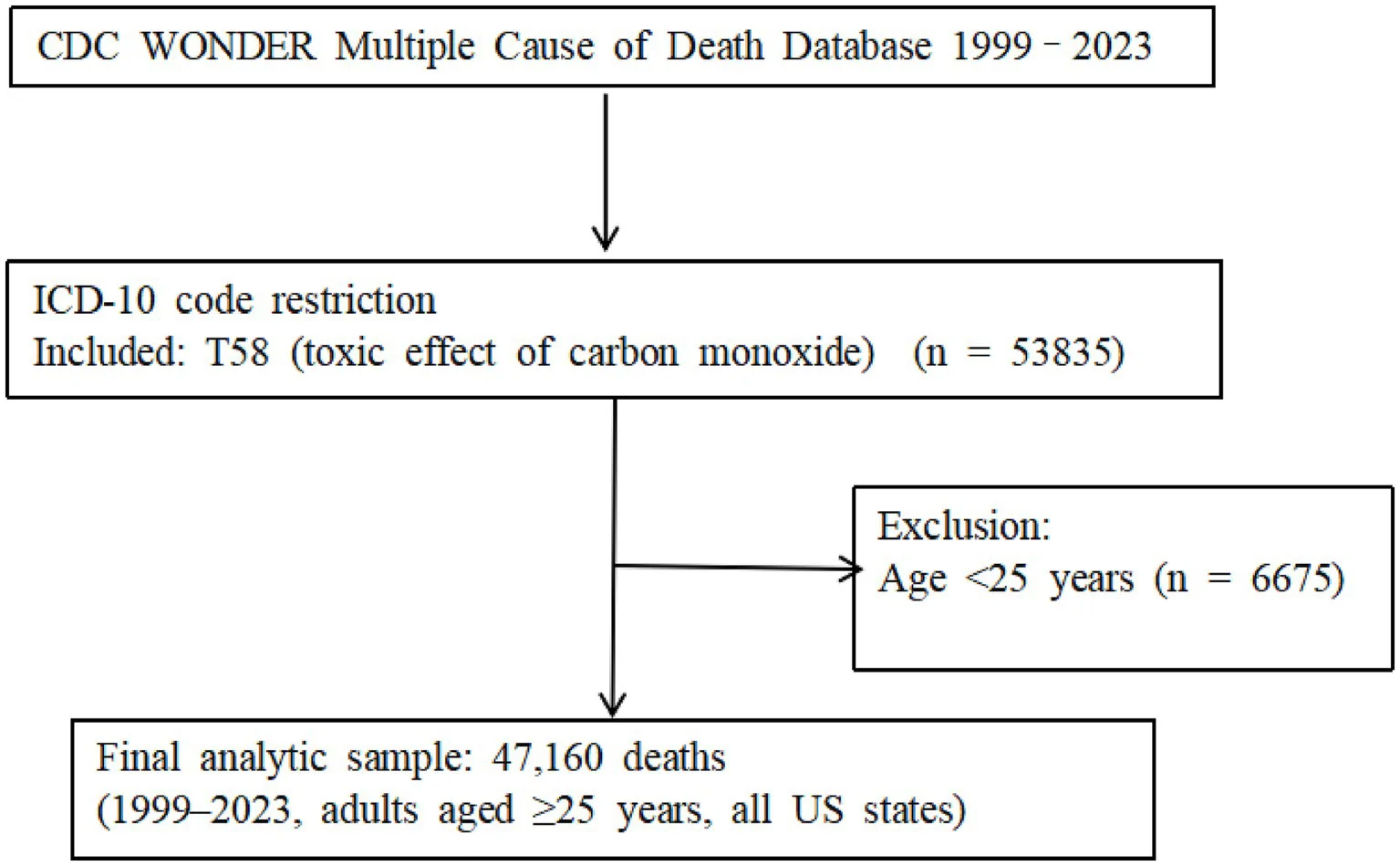
Flow diagram of sample selection for carbon monoxide (CO) poisoning–related mortality among adults aged ≥25 years, 1999–2023.

**Table 1:** Carbon monoxide poisoning-related deaths, age-adjusted mortality rates per 100,000 and average annual percent changes, stratified by sex, race and Census region in the United States, 1999 to 2023.

Subgroup	Deaths (1999)	Deaths (2023)	AAMR (1999)	AAMR (2023)	AAPC (1999–2023)
Overall	2,405	1,506	1.33 (1.27–1.38)	0.65 (0.61–0.68)	−3.02 (−4.58 to −1.43)*
Men	1,754	1,088	2.10 (2.00–2.20)	0.97 (0.91–1.03)	−3.17 (−4.48 to −1.84)*
Women	651	418	0.67 (0.62–0.72)	0.34 (0.31–0.38)	−2.80 (−4.14 to −1.44)*
NH White	2,067	1,076	1.53 (1.46–1.60)	0.72 (0.68–0.77)	−3.07 (−4.44 to −1.68)*
NH Black	212	173	1.15 (0.99–1.30)	0.64 (0.54–0.73)	−1.99 (−3.14 to −0.84)*
Hispanic	77	167	0.48 (0.37–0.63)	0.44 (0.37–0.50)	−0.83 (−2.06 to 0.43)
NH Other	49	90	0.48 (0.34–0.66)	0.38 (0.30–0.48)	−1.10 (−1.87 to −0.33)*
Northeast	351	202	0.99 (0.88–1.09)	0.49 (0.42–0.56)	−2.92 (−3.31 to −2.52)*
Midwest	815	495	2.00 (1.86–2.13)	1.02 (0.92–1.11)	−3.01 (−4.86 to −1.12)*
South	755	467	1.19 (1.10–1.27)	0.50 (0.46–0.55)	−3.76 (−4.13 to −3.39)*
West	484	342	1.23 (1.12–1.34)	0.64 (0.57–0.71)	−2.45 (−3.44 to −1.45)*
Metropolitan	1,946	1,148	1.29 (1.24–1.35)	0.57 (0.53–0.60)	−3.56 (−4.21 to −2.90)*
Non-metropolitan	459	329	1.58 (1.44–1.73)	1.02 (0.90–1.14)	−2.32 (−3.36 to −1.26)*
25–34 years	445	206	1.11 (1.11–1.11)	0.45 (0.45–0.45)	−3.90 (−4.95 to −2.83)*
35–44 years	648	267	1.44 (1.44–1.44)	0.60 (0.60–0.60)	−3.85 (−5.18 to −2.50)*
45–54 years	518	280	1.42 (1.42–1.42)	0.69 (0.69–0.69)	−2.94 (−3.81 to −2.05)*
55–64 years	279	313	1.17 (1.17–1.17)	0.75 (0.75–0.75)	−2.12 (−2.44 to −1.80)*
65–74 years	187	208	1.01 (1.01–1.01)	0.60 (0.60–0.60)	−2.31 (−2.81 to −1.81)*
75–84 years	221	144	1.81 (1.81–1.81)	0.78 (0.78–0.78)	−3.02 (−3.61 to −2.42)*
85 + years	107	88	2.58 (2.58–2.58)	1.42 (1.42–1.42)	−2.45 (−3.14 to −1.76)*

AAMR, age-adjusted mortality rates; AAPC, average annual percent change; NH, non-Hispanic. *p < 0.05 for the AAPC (95% CI excludes zero).

For metropolitan and non-metropolitan subgroups, the 2023 death counts correspond to 2020, as urban–rural analyses in CDC WONDER were restricted to 1999–2020.

**Table 2:** Annual percent change (APC) of carbon monoxide poisoning-related age-adjusted mortality rates per 100,000 in the United States, 1999 to 2023.

Year interval	APC (95% CI)
Overall	
1999–2007	−2.47 (−3.74 to −1.18)*
2007–2010	−9.70 (−20.83 to 2.99)
2010–2023	−1.75 (−2.50 to −1.00)*
Men	
1999 to 2007	−2.65 (−3.72 to −1.56)*
2007 to 2010	−9.08 (−18.56 to 1.51)
2010 to 2023	−2.08 (−2.71 to −1.45)*
Women	
1999 to 2007	−2.87 (−3.93 to −1.80)*
2007 to 2010	−8.67 (−18.38 to 2.20)
2010 to 2023	−1.35 (−1.99 to −0.71)*
NH White	
1999 to 2007	−1.93 (−3.03 to −0.81)*
2007 to 2010	−8.21 (−18.15 to 2.9)
2010 to 2023	−2.55 (−3.24 to −1.86)*
NH Black	
1999 to 2017	−3.66 (−4.43 to −2.89)*
2017 to 2023	3.20 (−1.22 to 7.82)
NH Other	
1999 to 2023	−1.10 (−1.87 to −0.33)*
Hispanic	
1999 to 2016	−4.31 (−5.45 to −3.16)*
2016 to 2023	8.18 (4.42 to 12.07)*
Non-metropolitan areas	
1999 to 2013	−3.51 (−4.40 to −2.61)*
2013 to 2020	0.11 (−2.80 to 3.11)
Metropolitan area	
1999 to 2006	−2.75 (−3.72 to −1.77)*
2006 to 2012	−6.30 (−8.05 to −4.51)*
2012 to 2020	−2.17 (−3.15 to −1.17)*

APC, annual percent change; NH, non-Hispanic. The symbol * indicates that the value (AAPC in this table) is statistically significant at *p* < 0.05, meaning the 95% confidence interval does not cross zero.

### Age-adjusted mortality by sex

Across the study period, men consistently exhibited higher age-adjusted mortality rates than women (2023: 0.97 per 100,000 vs. 0.34 per 100,000). Temporal patterns mirrored the overall national trend: an initial decline (1999–2007: −2.65% in men and −2.87% in women), a steeper decline (2007–2010: −9.08% in men and −8.67% in women), and a slower decline thereafter (2010–2023: −2.08% in men and −1.35% in women) (Table 2 and Figure 2). Over the full study period (1999–2023), the AAPC was −3.17% (95% CI: −4.48 to −1.84) for men and −2.80% (95% CI: −4.14 to −1.44) for women.

**Figure 2:**
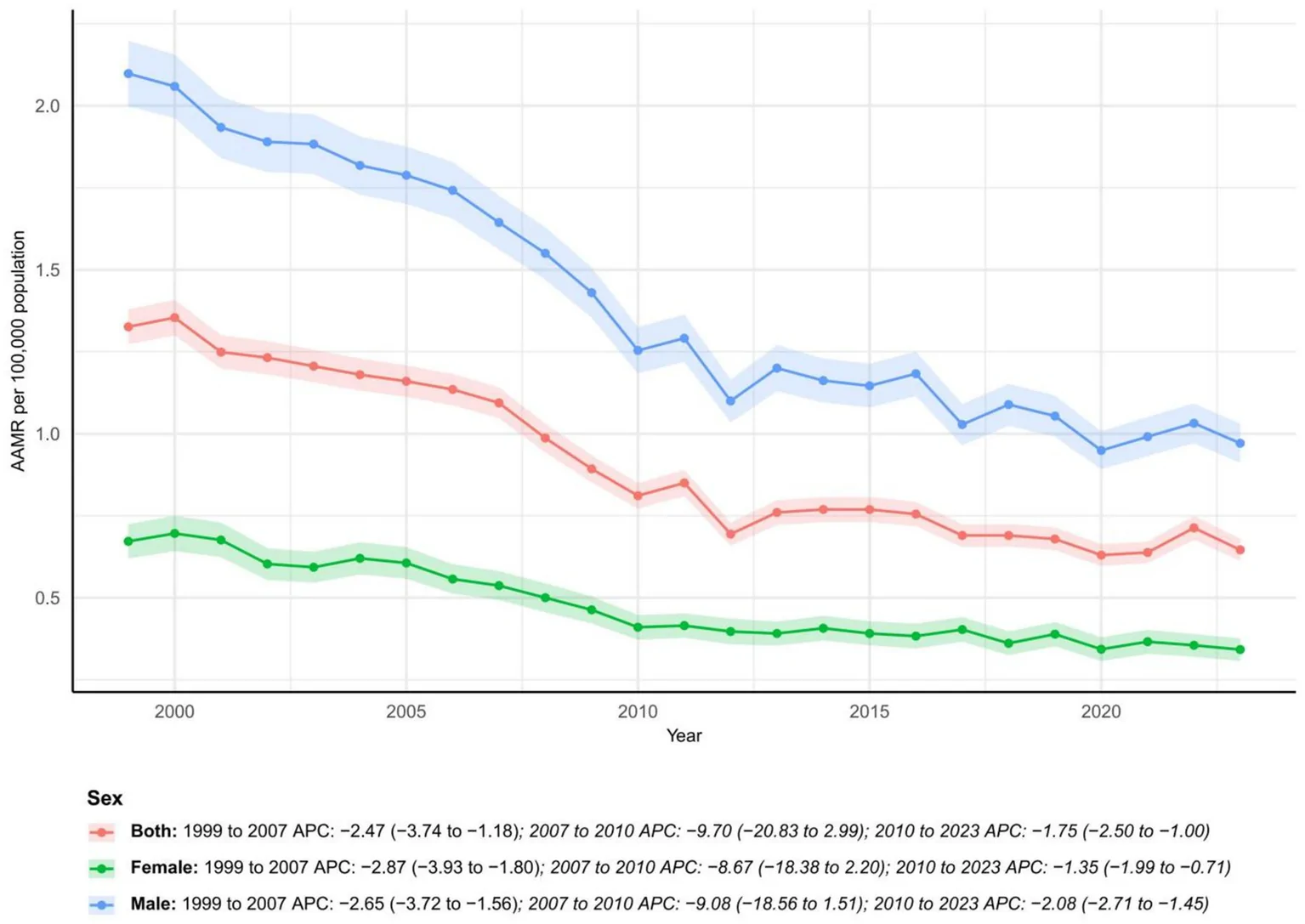
Trends in age-adjusted mortality rates for unintentional, non–fire-related carbon monoxide poisoning among adults aged 25 years and older, by sex, United States, 1999–2023.

### Age-adjusted mortality by race and ethnicity

Marked disparities and divergent trends were observed across racial and ethnic groups (Tables 1, 2). Non-Hispanic White adults had the highest overall mortality burden but experienced the steepest decline from 2010 to 2023 (APC: −2.55%; 95% CI: −3.24 to −1.86). In contrast, mortality among Hispanic adults reversed from a decline of −4.31% (1999–2016; Table 2) to a substantial increase of 8.18% (2016–2023; 95% CI: 4.42 to 12.07; Table 2). Non-Hispanic Black adults experienced a sustained decline from 1999 to 2017 (APC: −3.66%; 95% CI: −4.43 to −2.89; Table 2), followed by a non-significant reversal from 2017 to 2023 (APC: 3.20%; 95% CI: −1.22 to 7.82; Table 2), indicating a potential reversal of previous improvements (Figure 3). To assess whether the observed increase in Hispanic mortality was confounded by urban–rural status, we stratified Hispanic deaths by 2013 Urbanization. The majority of Hispanic CO poisoning deaths occurred in metropolitan areas: 2,636 of 2,988 (88.2%) during 1999–2020 and 1,085 of 1,209 (89.7%) during 2018–2023, with only 352 (11.8%) and 124 (10.3%) occurring in nonmetropolitan areas, respectively (Supplementary Table 3). Although the majority of Hispanic CO poisoning deaths occurred in metropolitan areas, this distribution does not exclude the possibility that Hispanic populations within urban settings may face distinct, localized risks—including substandard housing conditions, language barriers, and economic constraints—that could contribute to the observed mortality increase. Formal interaction testing between race/ethnicity and urban–rural status was not performed.

**Figure 3:**
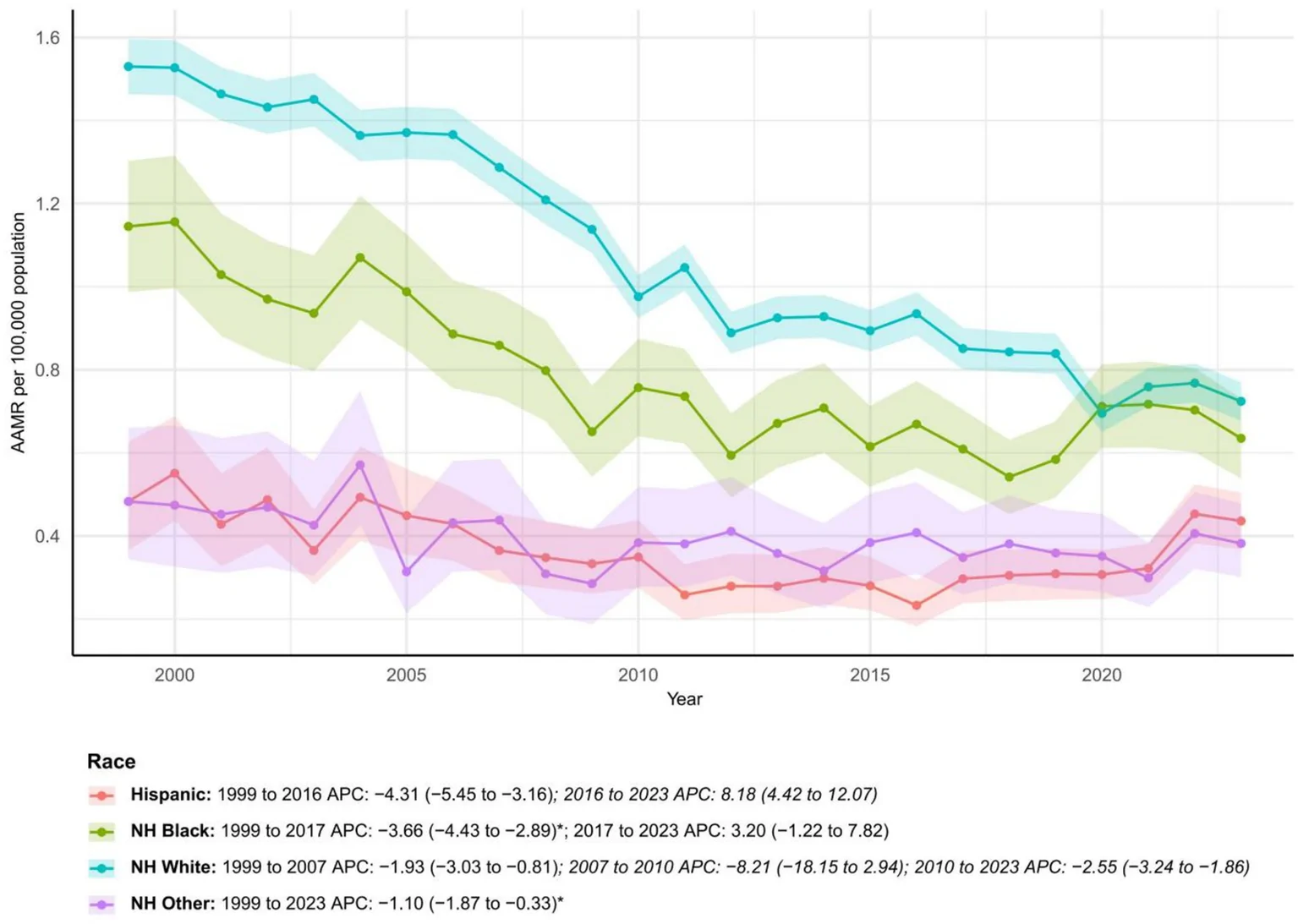
Trends in age-adjusted mortality rates for unintentional, non–fire-related carbon monoxide poisoning among adults aged 25 years and older, by race and ethnicity, United States, 1999–2023.

### Age-adjusted mortality by each age group

Between 1999 and 2023, all age groups experienced overall declines in age-adjusted mortality (AAPC range: −2.12 to −3.90%). Younger adults (25–34 and 35–44 years) showed faster initial declines, with a slower rate of decline in more recent years (Table 1 and Figure 4). The 25–34 age group had the fastest decline (AAPC: −3.90%; 95% CI: −4.95 to −2.83), whereas the 55–64 age group had the slowest decline (AAPC: −2.12%; 95% CI: −2.44 to −1.80) and was the only age group in which the absolute number of deaths increased (from 279 in 1999 to 313 in 2023).

**Figure 4:**
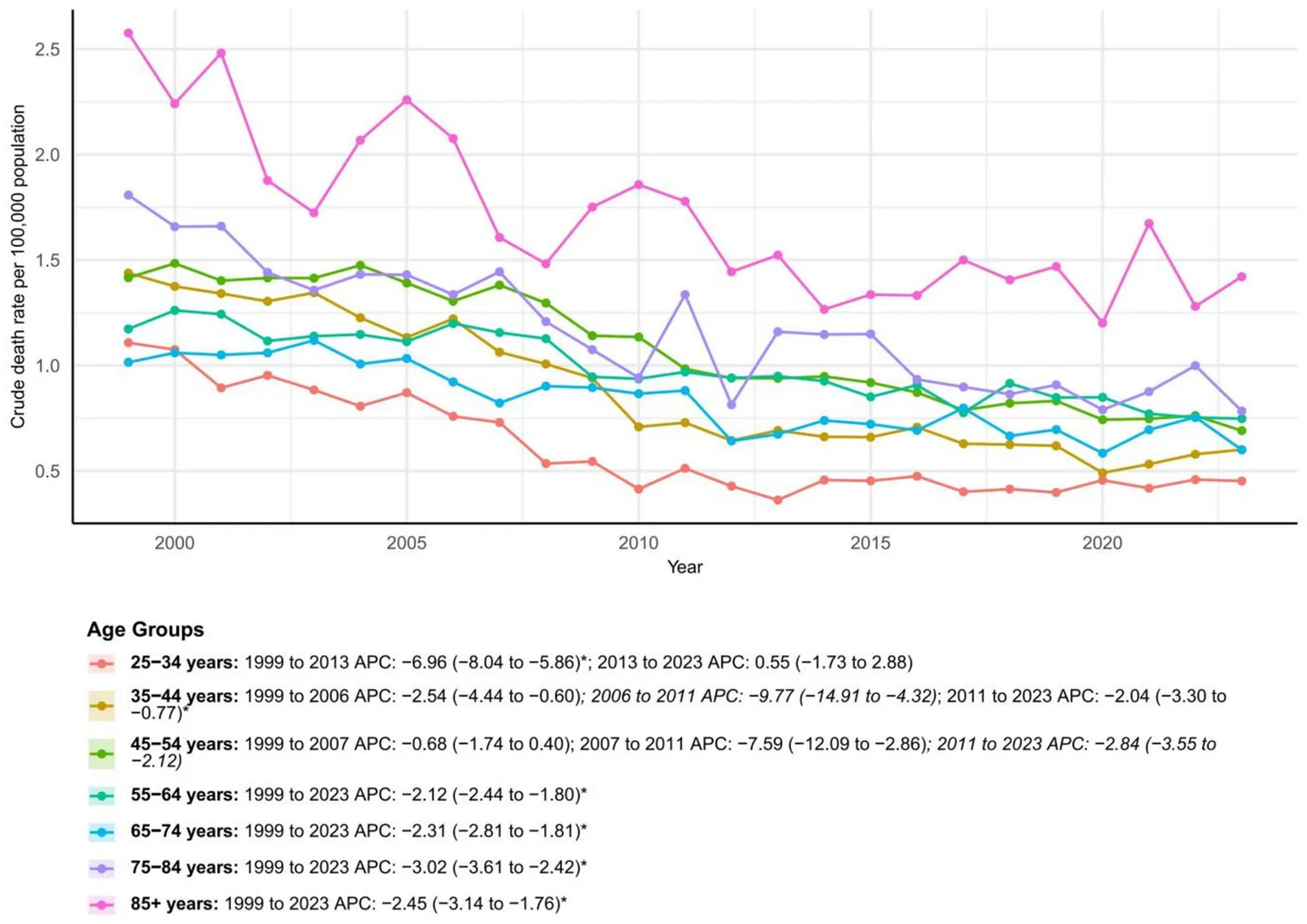
Trends in age-adjusted mortality rates for unintentional, non–fire-related carbon monoxide poisoning among adults aged 25 years and older, by age group, United States, 1999–2023.

### Age-adjusted mortality by geographic region

Substantial geographic variation was observed (Figure 5). The South (AAPC: −3.76%) and Midwest (AAPC: −3.01%) exhibited the fastest overall declines in CO poisoning mortality. In contrast, in the West, the rate of decline slowed after 2012, with no significant change from 2012 to 2023 (APC − 0.74%; 95% CI: −1.72 to 0.24). State-level analyses are presented in Figure 6 and Supplementary Table 4. In 2023, age-adjusted mortality ranged from 0.29 per 100,000 in North Carolina to 5.90 per 100,000 in Alaska. Alaska had the highest mortality rate (5.90 per 100,000), followed by Missouri (1.42 per 100,000), and Oklahoma (1.24 per 100,000) (Supplementary Table 4 and Figure 6B). From 1999 to 2023, percentage change in the number of deaths ranged from a 77% decrease in North Carolina to a 133% increase in Alaska (Supplementary Table 4 and Figure 6D).

**Figure 5:**
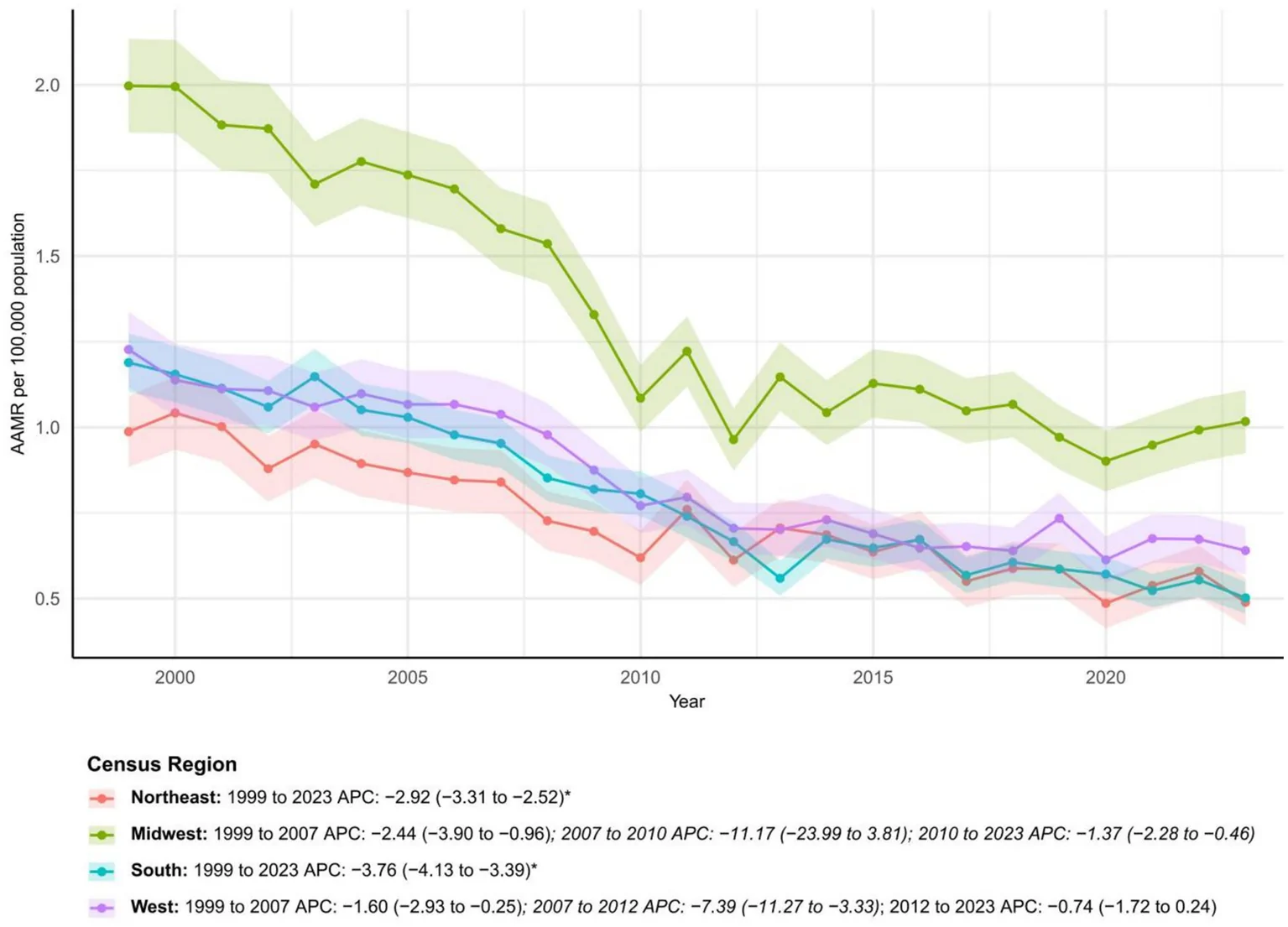
Trends in age-adjusted mortality rates for unintentional, non–fire-related carbon monoxide poisoning among adults aged 25 years and older, by U. S. Census region, United States, 1999–2023.

**Figure 6:**
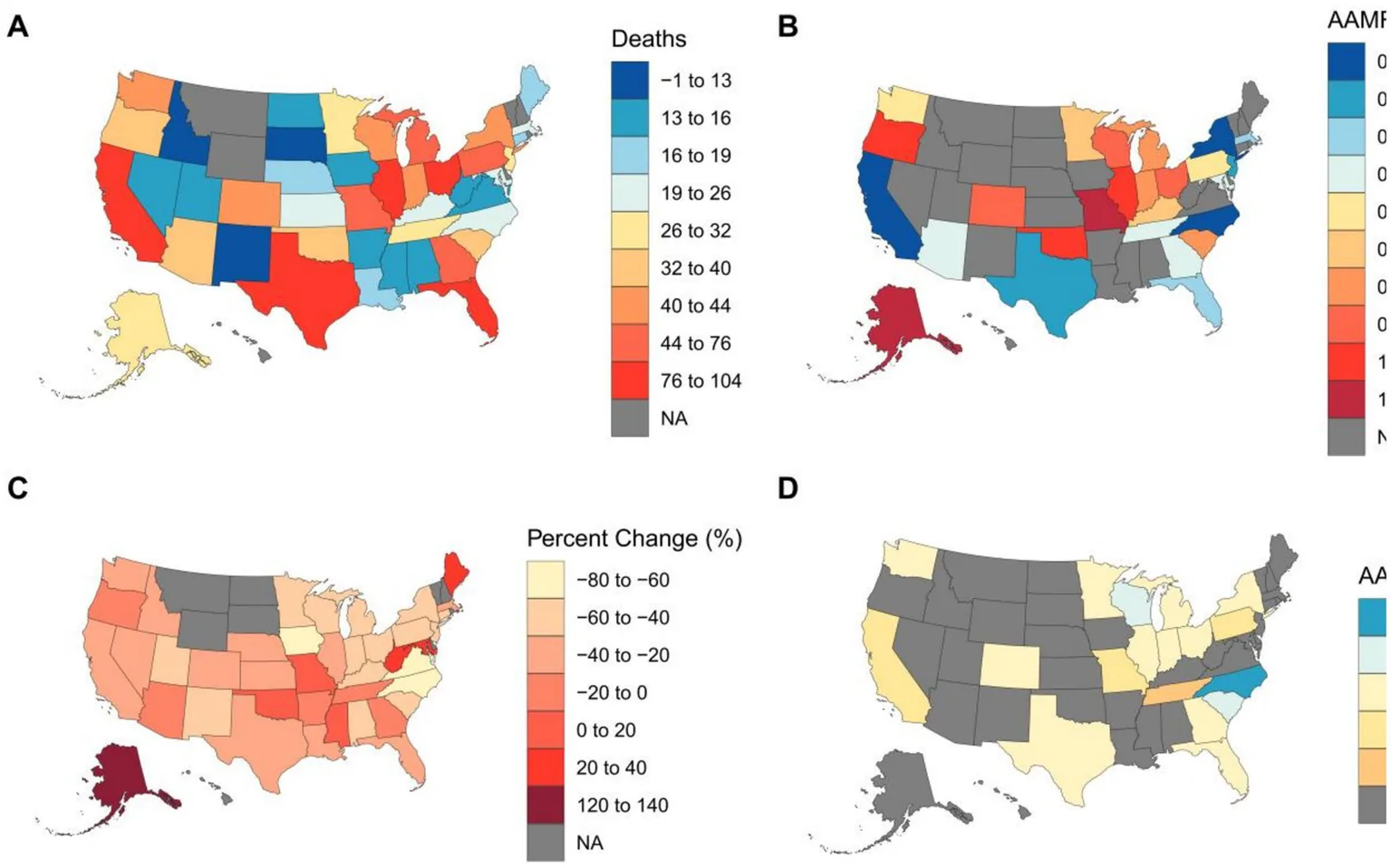
State-level variation in unintentional, non–fire-related carbon monoxide poisoning mortality among adults aged 25 years and older, United States, 1999–2023. **(A)** Total number of deaths in 2023. **(B)** Age-adjusted mortality rate (AAMR) per 100000 population in 2023. **(C)** Percent change in the number of deaths, 1999–2023. **(D)** Average annual percent change (AAPC) in the AAMR, 1999–2023.

### Age-adjusted mortality by urban–rural status

Mortality from CO poisoning was consistently higher in non-metropolitan than metropolitan areas (2023: 1.02 vs. 0.57 per 100,000). A divergence emerged in recent years: metropolitan areas experienced sustained declines from 2012 to 2020 (APC: −2.17%; 95% CI: −3.15 to −1.17), whereas mortality in non-metropolitan areas showed no significant change from 2013 to 2020 (APC: 0.11%; 95% CI: −2.80 to 3.11), suggesting a potential stall in progress in rural areas compared to earlier periods (Table 2).

## Discussion

This national analysis of mortality data from 1999 to 2023 reveals three core findings. First, after a prolonged period of decline, the overall age-adjusted CO poisoning mortality rate slowed markedly after 2010. Second, mortality among Hispanic adults reversed from a sustained decline to a substantial increase after 2016, with similar reversal signs emerging among non-Hispanic Black adults after 2017. Third, mortality in non-metropolitan areas stagnated while metropolitan areas continued to decline, widening the urban–rural gap.

Interpreting these patterns requires distinguishing between two mechanisms: changes in CO exposure incidence and changes in case fatality. While mortality data alone cannot separate these pathways, this framework helps generate hypotheses about the drivers of observed disparities. The three core findings may be interconnected—they may reflect the limits of universal prevention strategies in the face of increasingly unequal exposure risks, particularly among populations with differential vulnerability to extreme weather events, housing conditions, and healthcare access.

The slowing of the overall decline after 2010 likely reflects both case-fatality and incidence factors. On the case-fatality side, household detectors—the primary technical foundation of CO prevention—have notable reliability limitations. Ryan and Arnold (7) reported a detector failure rate of 57%, including 40% classified as critical “unsafe failures,” with performance strongly correlated with detector age. Public awareness and behavioral compliance are also limiting: in a Wisconsin survey, only 46% of respondents were aware of local CO alarm ordinances and 45% reported compliant installation (8). On the incidence side, extreme weather events and power outages have become more frequent, increasing CO exposure through generator use and alternative heating sources. The convergence of aging detectors and rising exposure risks may explain why the long-term decline has slowed despite continued prevention efforts.

Mortality has continued to decline among non-Hispanic White adults, whereas rates have increased substantially among Hispanic adults since 2016, and show signs of reversal among non-Hispanic Black adults. This widening gap may suggest that existing broad strategies may not have effectively reached or protected these communities. The widening disparities observed in this study may reflect broader structural factors—such as those related to housing quality, heating sources, or appliance safety—that have been previously associated with CO poisoning risk (9–11). However, because our data do not include individual-level socioeconomic information, we cannot directly attribute the observed trends to these specific mechanisms. Cultural and linguistic barriers may also contribute to disparities in the reach of safety communication and prevention efforts.

Sex differences are also noteworthy. Male mortality was approximately 2.9-fold higher than female mortality in 2023, consistent with prior literature (3, 6). Potential explanations include differential occupational exposure—men are more likely to work in generator operation, furnace maintenance, or automotive repair—as well as greater engagement in risk behaviors and potentially lower rates of detector maintenance or emergency care-seeking. Interestingly, despite higher absolute rates, male mortality declined faster over the study period (AAPC: −3.17% vs. −2.80% for women). This may reflect improvements in occupational safety standards, differential adoption of prevention measures, or a ceiling effect whereby populations with higher baseline rates have more room for improvement. However, as CDC WONDER data lack occupation or behavioral information, these mechanisms remain speculative.

Geographic disparities are substantial. Age-adjusted mortality in non-metropolitan areas remained approximately 1.8 times higher than in metropolitan areas. From 2012 to 2020, metropolitan areas continued to decline (APC: −2.17%; 95% CI: −3.15 to −1.17), whereas non-metropolitan areas showed no significant change (APC: 0.11%; 95% CI: −2.80 to 3.11), suggesting a stall in rural progress. This pattern likely reflects both incidence and case-fatality factors. Rural areas have greater reliance on higher-risk heating fuels and are more vulnerable to power outages (incidence), while also facing reduced access to emergency services, longer ambulance response times, and delayed dissemination of updated safety information (case fatality) (12, 13). Alaska exemplifies how multiple vulnerabilities—severe cold, off-grid living, and frequent power outages—can converge to create extreme risk (14, 15, 16).

Annual mortality data revealed recurrent upticks in certain years, suggesting episodic surges superimposed on a declining baseline. These clusters coincided with extreme winter events that caused widespread power outages and prompted indoor generator use—including 2011 (Hurricane Irene), 2014 (polar vortex), 2016 (Winter Storm Jonas), and 2022 (winter storms)—well-recognized drivers of CO poisoning outbreaks (17–19). Power loss drives reliance on backup generators and other combustion devices, often used in enclosed spaces, leading to acute CO exposure—a cascade documented following major hurricanes, including Katrina (2005) and Sandy (2012) (4). This pattern suggests that, despite overall decline, the U. S. population remains vulnerable to disaster-driven CO poisoning surges—a vulnerability that may warrant increased attention under climate change.

The study period also encompassed the COVID-19 pandemic (2020–2023), which may have influenced CO poisoning mortality through multiple pathways. Behavioral changes during this period—including increased time spent at home, pandemic-related economic stressors prompting reliance on alternative or portable heating sources, and greater use of generators during weather-related power outages—could have increased CO exposure risk, particularly among vulnerable populations. Concurrently, disruptions in healthcare access during the pandemic may have affected case fatality by delaying or reducing access to emergency services and hyperbaric oxygen therapy. In our data, the overall national mortality decline continued through 2023, with no clear interruption in the long-term trend. However, the pre-existing reversals among Hispanic adults (beginning in 2016) and non-Hispanic Black adults (beginning in 2017) warrant attention, as these groups experienced disproportionate COVID-19 burden, which may have exacerbated rather than initiated these trends. The pandemic period (2020–2023) may have amplified existing disparities through increased exposure risks and reduced healthcare access, particularly for these already-vulnerable populations. International evidence from Korea and Germany suggests that the pandemic was associated with increased intentional CO poisoning and prolonged time to hyperbaric oxygen therapy (20, 21), suggesting that both exposure patterns and treatment access may have shifted during this period. In our data, the reversals among Hispanic and non-Hispanic Black populations may reflect these pandemic-related vulnerabilities, but we cannot determine whether they were specifically attributable to the pandemic or reflected continuation of pre-existing patterns. Our descriptive trend analysis does not permit causal attribution of pandemic-period changes to specific mechanisms. Nor can we account for individual-level factors that may modify CO toxicity—such as inflammatory, metabolic, or antioxidant status (22)—as our data are ecological in nature and lack such host-level information.

These findings should be interpreted in the context of several limitations. Death certificate data are subject to misclassification and underreporting, and ICD coding practices may have changed over the study period. The use of Multiple Cause of Death data may include cases where CO exposure was incidental rather than causative and precludes exclusion of intentional or fire-related deaths. We also applied a fixed 2013 urban–rural classification across the entire period, potentially introducing temporal misclassification bias. Our analysis is restricted to mortality data and does not capture non-fatal CO poisoning events. In the absence of incidence data, we cannot determine whether observed trends reflect changes in exposure frequency, case fatality, or both. Additionally, rates in low-population states may be unstable; we addressed this by presenting 95% confidence intervals and suppressing estimates based on fewer than 10 deaths. Multiple subgroup comparisons were performed without adjustment for multiple testing, so some significant findings may be due to chance. Finally, the ecological nature of subgroup comparisons means that group-level associations may not hold at the individual level. Nonetheless, this national long-term analysis provides a clear delineation of the magnitude and multidimensional nature of CO poisoning disparities.

## Conclusion

The observed trends indicate that the long-term decline in CO poisoning mortality may have reached a turning point. The complex and unequal risk landscape observed in this study suggests that population-wide approaches alone may be insufficient to address persistent disparities. Our findings suggest that future research should investigate whether a transition from universal to more targeted strategies—incorporating technological reliability, public awareness, and differential access to prevention resources—could mitigate the observed disparities.

## Data Availability

The data analyzed in this study are publicly available from the CDC database. Further inquiries can be directed to the corresponding author.
