# Supplementary material for: Persistent disparities despite declining carbon monoxide mortality in the United States: a nationwide analysis, 1999–2023
Source: Front Public Health. 2026 Sep 22;14:1877803. doi: 10.3389/fpubh.2026.1877803 (PMC13638626; doi:10.3389/fpubh.2026.1877803)
Supplement: Supplementary file 1 [file Table_1.docx]

**Supplemental Table 1. CDC WONDER Data Extraction Protocol.**

| **Parameter** | **Setting** |
| --- | --- |
| Database | CDC WONDER Multiple Cause of Death |
| Years | 1999-2023 |
| ICD-10 codes | T58 |
| Age restriction | ≥25 years |
| Age standardization | Direct method, 2000 US standard population |
| Race/ethnicity categories | Non-Hispanic White, Non-Hispanic Black, Hispanic, Non-Hispanic Other |
| Urban-rural classification | 2013 NCHS Urban-Rural Classification Scheme |
| Data suppression | Rates based on <10 deaths not reported |
| Date of data extraction | [July 5, 2025] |

**Supplemental Table 2. Carbon monoxide poisoning-related Deaths, Stratified by Sex, Race and Census region in the United States, 1999 to 2023**

| Deaths | | | | | | | | | | | | |
| --- | --- | --- | --- | --- | --- | --- | --- | --- | --- | --- | --- | --- |
| Year | Overall | Women | Men | NH White | NH Black | NH other | Hispanic | Northeast | Midwest | South | West | Population |
| 1999 | 2,405 | 651 | 1,754 | 2,067 | 212 | 49 | 77 | 351 | 815 | 755 | 484 | 180,408,769 |
| 2000 | 2,410 | 652 | 1,758 | 2,055 | 213 | 43 | 99 | 365 | 827 | 765 | 453 | 181,984,640 |
| 2001 | 2,330 | 645 | 1,685 | 2,011 | 199 | 42 | 78 | 357 | 787 | 729 | 457 | 184,305,128 |
| 2002 | 2,282 | 604 | 1,678 | 1,948 | 191 | 53 | 90 | 323 | 787 | 714 | 458 | 186,208,028 |
| 2003 | 2,296 | 620 | 1,676 | 1,982 | 183 | 50 | 81 | 351 | 728 | 771 | 446 | 188,090,429 |
| 2004 | 2,272 | 625 | 1,647 | 1,893 | 209 | 73 | 97 | 331 | 751 | 729 | 461 | 190,205,384 |
| 2005 | 2,255 | 619 | 1,636 | 1,914 | 201 | 44 | 96 | 330 | 739 | 735 | 451 | 192,551,384 |
| 2006 | 2,228 | 608 | 1,620 | 1,886 | 187 | 49 | 106 | 324 | 739 | 701 | 464 | 195,019,359 |
| 2007 | 2,167 | 583 | 1,584 | 1,828 | 188 | 57 | 94 | 317 | 696 | 691 | 463 | 197,403,777 |
| 2008 | 2,021 | 525 | 1,496 | 1,716 | 175 | 44 | 86 | 279 | 666 | 630 | 446 | 199,795,090 |
| 2009 | 1,883 | 506 | 1,377 | 1,617 | 142 | 37 | 87 | 271 | 592 | 614 | 406 | 202,107,016 |
| 2010 | 1,727 | 472 | 1,255 | 1,422 | 168 | 51 | 86 | 243 | 496 | 616 | 372 | 203,891,983 |
| 2011 | 1,795 | 493 | 1,302 | 1,503 | 170 | 53 | 69 | 301 | 561 | 562 | 371 | 206,592,936 |
| 2012 | 1,568 | 437 | 1,131 | 1,302 | 140 | 56 | 70 | 243 | 459 | 519 | 347 | 208,826,037 |
| 2013 | 1,637 | 453 | 1,184 | 1,350 | 160 | 53 | 74 | 287 | 529 | 479 | 342 | 211,085,314 |
| 2014 | 1,680 | 476 | 1,204 | 1,365 | 176 | 53 | 86 | 280 | 490 | 558 | 352 | 213,809,280 |
| 2015 | 1,656 | 463 | 1,193 | 1,350 | 161 | 60 | 85 | 258 | 519 | 533 | 346 | 216,553,817 |
| 2016 | 1,663 | 435 | 1,228 | 1,359 | 165 | 63 | 76 | 262 | 524 | 528 | 349 | 218,641,417 |
| 2017 | 1,565 | 476 | 1,089 | 1,241 | 165 | 62 | 97 | 219 | 501 | 496 | 349 | 221,447,331 |
| 2018 | 1,604 | 420 | 1,184 | 1,288 | 150 | 62 | 104 | 242 | 504 | 506 | 352 | 223,311,190 |
| 2019 | 1,602 | 458 | 1,144 | 1,272 | 164 | 64 | 102 | 251 | 452 | 512 | 387 | 224,981,167 |
| 2020 | 1,477 | 388 | 1,089 | 1,097 | 204 | 65 | 111 | 181 | 429 | 534 | 333 | 226,635,013 |
| 2021 | 1,531 | 427 | 1,104 | 1,146 | 196 | 70 | 119 | 232 | 466 | 468 | 365 | 228,238,412 |
| 2022 | 1,600 | 435 | 1,165 | 1,156 | 192 | 88 | 164 | 242 | 477 | 506 | 375 | 229,508,599 |
| 2023 | 1,506 | 418 | 1,088 | 1,076 | 173 | 90 | 167 | 202 | 495 | 467 | 342 | 231,529,762 |
| **Total** | 47,160 | 12,889 | 34,271 | 38,844 | 4,484 | 1,431 | 2,401 | 7,042 | 15,029 | 15,118 | 9,971 | 5,163,131,262 |

NH, non-Hispanic;

**Supplemental Table 3. Number of Carbon Monoxide Poisoning Deaths by Hispanic Origin, Race, and Urban–Rural Status, United States, 1999–2023.**

| **Hispanic Origin** | **Race** | **2013 Urbanization** | **1999–2020 Deaths** | **2018–2023 Deaths** |
| --- | --- | --- | --- | --- |
| Hispanic or Latino | White | Large Central Metro | 1,154 | 486 |
|  | White | Large Fringe Metro | 533 | 218 |
|  | White | Medium Metro | 687 | 287 |
|  | White | Small Metro | 224 | 94 |
|  | White | Micropolitan (Nonmetro) | 237 | 81 |
|  | White | NonCore (Nonmetro) | 115 | 43 |
|  | Black or African American | Large Central Metro | 27 | — |
|  | Black or African American | Medium Metro | — | — |
|  | Black or African American | Micropolitan (Nonmetro) | — | — |
|  | Asian or Pacific Islander | Medium Metro | 11 | — |
|  | Asian | Small Metro | — | — |
| Not Hispanic or Latino | White | Large Central Metro | 7,871 | 1,516 |
|  | White | Large Fringe Metro | 10,145 | 2,238 |
|  | White | Medium Metro | 10,400 | 2,260 |
|  | White | Small Metro | 5,440 | 1,238 |
|  | White | Micropolitan (Nonmetro) | 5,586 | 1,391 |
|  | White | NonCore (Nonmetro) | 4,194 | 1,041 |
|  | Black or African American | Large Central Metro | 1,995 | 547 |
|  | Black or African American | Large Fringe Metro | 841 | 257 |
|  | Black or African American | Medium Metro | 1,125 | 283 |
|  | Black or African American | Small Metro | 450 | 140 |
|  | Black or African American | Micropolitan (Nonmetro) | 378 | 90 |
|  | Black or African American | NonCore (Nonmetro) | 345 | 79 |
|  | American Indian or Alaska Native | Large Central Metro | 51 | 20 |
|  | American Indian or Alaska Native | Large Fringe Metro | 38 | — |
|  | American Indian or Alaska Native | Medium Metro | 89 | 22 |
|  | American Indian or Alaska Native | Small Metro | 75 | 22 |
|  | American Indian or Alaska Native | Micropolitan (Nonmetro) | 144 | 40 |
|  | American Indian or Alaska Native | NonCore (Nonmetro) | 167 | 64 |
|  | Asian or Pacific Islander | Large Central Metro | 423 | 141 |
|  | Asian or Pacific Islander | Large Fringe Metro | 184 | 64 |
|  | Asian or Pacific Islander | Medium Metro | 169 | 47 |
|  | Asian or Pacific Islander | Small Metro | 46 | 11 |
|  | Asian or Pacific Islander | Micropolitan (Nonmetro) | 30 | — |
|  | More than one race | Large Central Metro | — | 30 |
|  | More than one race | Large Fringe Metro | — | 30 |
|  | More than one race | Medium Metro | — | 23 |
|  | More than one race | Small Metro | — | 13 |
|  | More than one race | Micropolitan (Nonmetro) | — | 19 |

**Supplemental Table 4.** Carbon Monoxide Poisoning–Related Deaths and Age-Adjusted Mortality Rates per 100,000, Stratified by States in the United States, 1999–2023.

| State | Deaths | | | AAMR | | |
| --- | --- | --- | --- | --- | --- | --- |
|  | 1999 | 2023 | Percent.change | 1999 | 2023 | AAPC (95% CI) |
| Alabama | 26 | 15 | -42.31 | 0.94 (0.62 to 1.39) | NA (0.22 to 0.70) | - |
| Alaska | 12 | 28 | 133.33 | NA (1.79 to 8.16) | 5.90 (3.89 to 8.59) | - |
| Arizona | 38 | 32 | -15.79 | 1.23 (0.87 to 1.69) | 0.60 (0.40 to 0.85) | - |
| Arkansas | 17 | 15 | -11.76 | NA (0.59 to 1.62) | NA (0.42 to 1.25) | - |
| California | 142 | 102 | -28.17 | 0.69 (0.57 to 0.80) | 0.37 (0.29 to 0.44) | -2.25 (-6.60 to 2.31) |
| Colorado | 63 | 43 | -31.75 | 2.31 (1.77 to 2.96) | 1.07 (0.77 to 1.44) | -3.83 (-4.58 to -3.06)* |
| Connecticut | 32 | 17 | -46.88 | 1.43 (0.98 to 2.02) | NA (0.35 to 0.99) | - |
| Delaware | - | - | NA | NA (NA to NA) | NA (NA to NA) | - |
| District of Columbia | - | - | NA | NA (0.00 to 0.00) | NA (0.00 to 0.00) | - |
| Florida | 138 | 88 | -36.23 | 1.28 (1.07 to 1.50) | 0.52 (0.41 to 0.64) | -3.63 (-4.80 to -2.44)* |
| Georgia | 51 | 44 | -13.73 | 1.00 (0.74 to 1.32) | 0.60 (0.43 to 0.80) | -3.29 (-4.66 to -1.90)* |
| Hawaii | - | - | NA | NA (NA to NA) | NA (NA to NA) | - |
| Idaho | 17 | 11 | -35.29 | NA (1.28 to 3.52) | NA (0.42 to 1.60) | - |
| Illinois | 163 | 103 | -36.81 | 2.08 (1.76 to 2.41) | 1.22 (0.98 to 1.47) | -3.65 (-4.48 to -2.82)* |
| Indiana | 83 | 42 | -49.40 | 2.14 (1.70 to 2.65) | 0.88 (0.63 to 1.20) | -3.96 (-4.80 to -3.11)* |
| Iowa | 44 | 13 | -70.45 | 2.33 (1.70 to 3.13) | NA (0.26 to 0.88) | - |
| Kansas | 30 | 19 | -36.67 | 1.78 (1.20 to 2.54) | NA (0.57 to 1.51) | - |
| Kentucky | 44 | 25 | -43.18 | 1.70 (1.24 to 2.29) | 0.81 (0.52 to 1.21) | - |
| Louisiana | 25 | 17 | -32.00 | 0.92 (0.60 to 1.36) | NA (0.30 to 0.84) | - |
| Maine | 13 | 16 | 23.08 | NA (0.79 to 2.54) | NA (0.82 to 2.52) | - |
| Maryland | 18 | 23 | 27.78 | NA (0.30 to 0.78) | 0.55 (0.34 to 0.83) | - |
| Massachusetts | 34 | 22 | -35.29 | 0.80 (0.56 to 1.12) | 0.45 (0.28 to 0.69) | - |
| Michigan | 122 | 64 | -47.54 | 1.92 (1.58 to 2.27) | 0.86 (0.66 to 1.11) | -3.60 (-4.57 to -2.62)* |
| Minnesota | 67 | 31 | -53.73 | 2.14 (1.66 to 2.72) | 0.81 (0.55 to 1.17) | -3.09 (-3.79 to -2.39)* |
| Mississippi | 13 | 13 | 0.00 | NA (0.40 to 1.30) | NA (0.32 to 1.06) | - |
| Missouri | 60 | 62 | 3.33 | 1.70 (1.30 to 2.19) | 1.42 (1.07 to 1.84) | -2.37 (-3.27 to -1.46)* |
| Montana | 26 | - | NA | 4.50 (2.94 to 6.59) | NA (NA to NA) | - |
| Nebraska | 26 | 18 | -30.77 | 2.39 (1.56 to 3.51) | NA (0.90 to 2.40) | - |
| Nevada | 23 | 15 | -34.78 | 1.80 (1.14 to 2.71) | NA (0.38 to 1.16) | - |
| New Hampshire | - | - | NA | NA (NA to NA) | NA (NA to NA) | - |
| New Jersey | 51 | 29 | -43.14 | 0.91 (0.68 to 1.20) | 0.40 (0.26 to 0.58) | - |
| New Mexico | 28 | 12 | -57.14 | 2.49 (1.66 to 3.60) | NA (0.41 to 1.48) | - |
| New York | 85 | 40 | -52.94 | 0.68 (0.54 to 0.84) | 0.27 (0.19 to 0.37) | -3.22 (-3.90 to -2.54)* |
| North Carolina | 100 | 23 | -77.00 | 1.95 (1.57 to 2.34) | 0.29 (0.18 to 0.45) | -6.37 (-7.19 to -5.54)* |
| North Dakota | - | 13 | NA | NA (NA to NA) | NA (1.27 to 4.31) | - |
| Ohio | 128 | 76 | -40.62 | 1.73 (1.43 to 2.03) | 0.92 (0.72 to 1.16) | -3.42 (-4.21 to -2.63)* |
| Oklahoma | 33 | 33 | 0.00 | 1.52 (1.05 to 2.13) | 1.24 (0.85 to 1.75) | - |
| Oregon | 35 | 32 | -8.57 | 1.54 (1.07 to 2.14) | 1.09 (0.73 to 1.55) | - |
| Pennsylvania | 116 | 64 | -44.83 | 1.40 (1.14 to 1.65) | 0.66 (0.50 to 0.85) | -2.44 (-3.83 to -1.03)* |
| Rhode Island | 11 | - | NA | NA (0.78 to 2.81) | NA (NA to NA) | - |
| South Carolina | 56 | 32 | -42.86 | 2.21 (1.67 to 2.87) | 0.87 (0.58 to 1.24) | -4.30 (-5.26 to -3.32)* |
| South Dakota | - | 11 | NA | NA (NA to NA) | NA (1.04 to 3.72) | - |
| Tennessee | 33 | 28 | -15.15 | 0.90 (0.62 to 1.27) | 0.60 (0.39 to 0.87) | -1.16 (-2.57 to 0.27) |
| Texas | 121 | 77 | -36.36 | 0.98 (0.81 to 1.16) | 0.39 (0.31 to 0.49) | -3.36 (-4.87 to -1.82)* |
| Utah | 30 | 13 | -56.67 | 2.56 (1.73 to 3.65) | NA (0.36 to 1.15) | - |
| Vermont | - | - | NA | NA (NA to NA) | NA (NA to NA) | - |
| Virginia | 61 | 15 | -75.41 | 1.35 (1.03 to 1.74) | NA (0.15 to 0.46) | - |
| Washington | 52 | 41 | -21.15 | 1.35 (1.01 to 1.77) | 0.78 (0.56 to 1.07) | -3.39 (-4.16 to -2.61)* |
| West Virginia | 10 | 13 | 30.00 | NA (0.38 to 1.44) | NA (0.58 to 1.96) | - |
| Wisconsin | 78 | 43 | -44.87 | 2.27 (1.80 to 2.84) | 0.90 (0.64 to 1.24) | -4.62 (-5.58 to -3.64)* |
| Wyoming | 12 | - | NA | NA (1.97 to 6.65) | NA (NA to NA) | - |

AAMR, Age-Adjusted Mortality Rates; AAPC, Average Annual Percent Change; NH, non-Hispanic;NA, not available; -, not shown for fewer than 10 deaths; estimates are suppressed to ensure statistical reliability.
